# Association between patients with migraine and sarcopenia: A retrospective study

**DOI:** 10.1097/MD.0000000000038941

**Published:** 2024-07-12

**Authors:** Dong Ah Lee, Ho-Joon Lee, Jinseung Kim, Kang Min Park

**Affiliations:** a Department of Neurology, Haeundae Paik Hospital, Inje University College of Medicine, Busan, Republic of Korea; b Department of Radiology, Haeundae Paik Hospital, Inje University College of Medicine, Busan, Republic of Korea; c Department of Family Medicine, Busan Paik Hospital, Inje University College of Medicine, Busan, Republic of Korea.

**Keywords:** magnetic resonance imaging, migraine, sarcopenia

## Abstract

Recently, interest in sarcopenia has been increasing in patients with various neurological diseases. Thus, we investigated the presence of sarcopenia in patients with episodic migraine (EM) based on temporal muscle thickness (TMT). This was a retrospectively observational study following STROBE guidelines. We enrolled patients with EM and healthy controls. Both groups underwent brain magnetic resonance imaging, including three-dimensional T1-weighted imaging. We calculated the TMT using T1-weighted imaging, which is a marker for sarcopenia. We compared TMT between patients with EM and healthy controls, and analyzed it according to presence of migraine aura. We retrospectively enrolled 82 patients with EM and 53 healthy controls. TMT was not different between patients with EM and healthy controls (10.804 ± 2.045 mm in patients with EM vs 10.721 ± 1.547 mm in healthy controls, *P* = .801). Furthermore, TMT was not different according to presence of migraine aura in patients with EM (10.994 ± 2.016 mm in patients with migraine aura vs 10.716 ± 2.071 mm in those without, *P* = .569). There were no correlations between TMT and clinical characteristics in patients with EM, including age, age of onset, duration of migraine, headache intensity, and headache frequency. This study found no statistical difference in TMT between patients with EM and healthy controls or between patients with EM with and without aura. These findings suggest that there is no evidence of sarcopenia in patients with EM.

## 1. Introduction

Migraine is the most prevalent neurovascular brain disorder, impacting approximately 15% of the general population.^[1,2]^ The manifestations, characteristics, severity, duration, frequency, and associated features of migraine attacks exhibit considerable variability among individuals, partly influenced by sex.^[1]^ Despite advancements in experimental and clinical research contributing to our understanding of the neurobiological basis of migraine, the pathogenesis of this condition remains inadequately understood.^[3]^

The recently highlighted pathophysiological model of migraine revolves around neuroinflammation at the molecular level.^[4,5]^ Neuroinflammation denotes a broad immune response within the central nervous system, linked not only to migraine but also to various pain disorders. It encompasses increased vascular permeability, leukocyte infiltration, and activation of glial cells, leading to heightened production of inflammatory cytokines.^[4]^ However, there is little evidence supporting its role in the acute phase of migraine. Consequently, the pathophysiology of migraines involves a more localized form of neurogenic neuroinflammation, referring to the inflammatory reaction in specific parts of the trigeminovascular system.^[4]^ During migraine attacks, elevated levels of calcitonin gene-related peptide (CGRP) persist for up to 72 hours, potentially causing continuous activation of C and Aδ neurons.^[6–8]^ This prolonged activation is believed to induce trigeminal sensitization through the release of inflammatory cytokines near neuronal cell bodies in the trigeminal ganglion.^[7]^ Recently, various neuroimaging modalities have been employed to reveal neuroinflammation.^[9,10]^ According to a systematic review, macrophage activation is not observed in patients with migraine without aura, whereas migraine with aura shows evidence of microglial and parameningeal inflammatory activity. Increased vascular permeability, notably in hemiplegic migraine, is a focus of neuroinflammation studies, but the tools employed may not be optimal based on existing and emerging data.^[9]^ OnabotulinumtoxinA and CGRP monoclonal antibodies have established efficacy as standard preventive treatments for migraine, acting not only through the inhibition of sensory nerve endings via their anti-inflammatory effects but also by preventing the activation and sensitization of central neurons postulated to be involved in migraine chronification.^[11,12]^

Recently, interest in sarcopenia has been increasing in various neurological diseases. Sarcopenia is diagnosed as severe when it fulfills the operational definition of low muscle strength, low muscle quantity or quality, and low physical performance.^[13]^ Factors contributing to and exacerbating sarcopenia include aging, inflammatory conditions, neurological disorders, sedentary behavior, malnutrition, and iatrogenic conditions.^[13,14]^ Although the pathogenesis of sarcopenia is not fully understood, mitochondrial dysfunction, neuromuscular signaling, endocrine factors, and inflammation play crucial roles in its development.^[14]^ In this context, sarcopenia is hypothesized to play a significant pathophysiological role not only in the muscles but also within certain aspects of the nervous system. Significant associations between sarcopenia and neurological disorders, such as ischemic stroke, Parkinson’s disease, cognitive impairment, depression, and sleep disorders have been identified.^[15]^ Inflammation is the common mechanism underlying the occurrence of sarcopenia associated with various neurological diseases, with interleukin (IL)-1, IL-6, and tumor necrosis factor (TNF) identified as markers related to this process.^[15]^ These inflammatory markers are also major cytokines associated with the pathophysiology of migraine.^[4,16]^ Furthermore, evidence has demonstrated that the concentration of CGRP increases post-synaptically in animal experimental models with aging, resulting in changes in maintenance of neuromuscular junctions.^[17]^ Thus, there may be an association between patients with migraine and sarcopenia.

Sarcopenia cannot be diagnosed through one biomarker, necessitating the assessment of skeletal muscle strength through grip strength or the chair stand test, and the measurement of skeletal muscle mass through modalities such as dual-energy X-ray absorptiometry, magnetic resonance imaging (MRI), or computed tomography. Physical performance is evaluated using parameters such as gait speed and the timed up-and-go test.^[13,14]^ In a recent study, Craniofacial muscle mass has been identified as a reliable indicator for detecting sarcopenia, in addition to thoracolumbar skeletal muscle mass.^[18]^ In addition, a correlation between temporal muscle thickness (TMT), grip strength measurements, and body mass index was demonstrated.^[19]^ TMT was obtained from routinely conducted brain MRI and has been shown to estimate skeletal muscle mass.^[18,20]^ Recent research has confirmed a correlation between TMT and skeletal appendicular muscle mass in patients with Alzheimer’s disease. It was found that TMT was negatively correlated with age and positively correlated with body mass index. These findings suggest that TMT may be helpful in diagnosing sarcopenia.^[21]^

In this study, we explored the association between patients with migraine and sarcopenia using TMT measurement. From the perspective of neuroinflammation, we anticipated shared mechanisms between migraine and sarcopenia. Therefore, we hypothesized that migraine patients would be more likely to have sarcopenia compared to healthy controls.

## 2. Methods

### 2.1. Participants

This research was retrospectively conducted at a single specialized headache center. We obtained the requisite approval from the institutional review board at our hospital. Written informed consent to participate in this study was waived by the institutional review board. Our study involved the enrollment of patients who met the following criteria for migraine: newly diagnosed patients with episodic migraine (EM) with and without aura at our hospital, in accordance with the International Classification of Headache Disorders, 3rd edition^[22]^; patients who had undergone brain MRI, including three-dimensional T1-weighted imaging, at the time of their diagnosis of migraine; no structural brain lesions in their MRI; and absence of any medical or neurological disorders, except for EM. We conducted investigations into the demographic and clinical characteristics of the enrolled patients with EM, including age, sex, age of headache onset, duration of disease (measured as the time between the age of headache onset and MRI), monthly frequency of headache attacks, presence of aura, and headache intensity as assessed using a visual analog scale. Furthermore, we enrolled a control group comprising individuals matched for age and sex who were in good health and did not exhibit any medical or neurological disorders, especially EM. These participants had undergone normal brain MRI without any reported structural abnormalities.

### 2.2. MRI acquisition

Both patients with EM and the healthy control group underwent brain MRI using the same protocols performed on a 3-T MRI scanner equipped with a 32-channel head coil (AchievaTx; Phillips Healthcare, Best, Netherlands). The MRI sequences included 3D fluid-attenuated inversion recovery, coronal T2-weighted imaging, and 3D T1-weighted imaging, which are the standard MRI protocols employed for patients with EM at our hospital. To acquire the 3D T1-weighted images, a turbo-field echo sequence was utilized with the following parameters: TI = 1300 ms, repetition time/echo time = 8.6/3.96 ms, flip angle = 8°, and a voxel size of 1 mm^3^, ensuring uniform resolution in all dimensions.

### 2.3. TMT measurement

**Figure 1.**
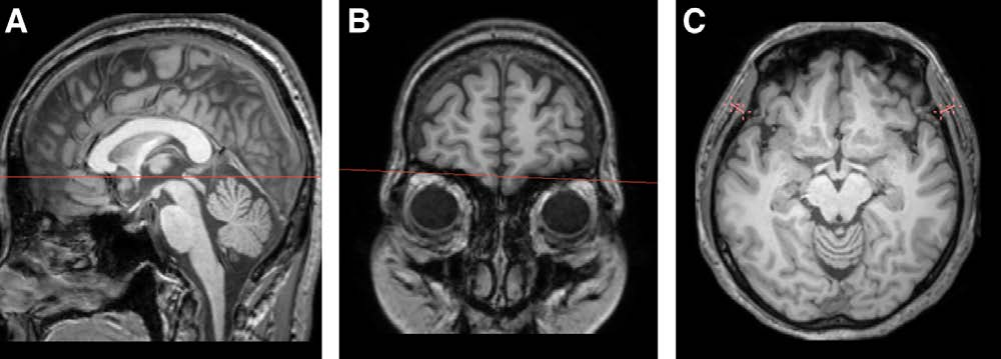
An example of the temporal muscle thickness measurement. (A) Images are reformatted to the axial plane parallel to the anterior commissure-posterior commissure line. (B) The view is navigated to the orbital roof level. (B) Thickness measurements of the temporalis muscle are taken on both sides, using the Sylvian fissure as an anterior-posterior reference point.

TMT was measured on 3D T1-weighted images on the right and left sides by a board-certified radiologist (HJL) with 9 years of subspecialty experience in neuroradiology. The images were reformatted to an axial plane parallel to the anterior commissure-posterior commissure line. Thereafter, TMT was measured perpendicular to the long axis of the temporalis muscle, using the orbital roof and the Sylvian fissure as landmarks. Image reformatting and measurements were performed using 3D Slicer (version 5.4.0, https://www.slicer.org).^[23,24]^ The measurements for each side were averaged for use in further analysis. Figure 1 illustrates the TMT measurement procedure.

### 2.4. Statistical analysis

We employed the independent-samples *t* test to compare age and TMT between the groups, while Fisher’s exact test was utilized for analyzing sex differences. Age of onset, duration of migraine, headache intensity, and headache frequency were compared between EM patients with and without aura using the Mann–Whitney *U* test. For correlation analysis between TMT and clinical factors, we employed the Pearson correlation test. All statistical analyses were executed using MedCalc software (version 20.014; MedCalc Software, Ostend, Belgium; accessible at https://www.medcalc.org; 2021). Statistical significance was established at a threshold of *P* < .05.

## 3. Results

### 3.1. Clinical characteristics in participants

We enrolled 82 patients with EM and 53 healthy controls. Table 1 shows the clinical characteristics of patients with EM and healthy controls. Age and sex were not significantly different among groups. Of the 82 patients with EM, 26 had migraine aura, whereas 56 did not. There were significantly more males with EM and aura than those without aura (8/26 [31%] vs 7/56 [13%], *P* = .047). Migraine attacks were more frequent in patients without aura than in those with aura (2 vs 4 per month, *P* = .007). There were no significant differences in other clinical migraine characteristics between patients with and without aura.

**Table 1:** Clinical characteristics in participants.

	Patients with episodic migraine (N = 82)	Healthy controls (N = 53)	*P* value
Age, years (±standard deviation)	30.6 (±10.2)	29.2 (±4.4)	.334
Male, n (%)	15 (18.3)	9 (17.0)	.846
Age of headache onset, years (interquartile range)	20 (12–30)		
Disease duration, months (interquartile range)	60 (24–120)		
Attack frequency per month, n (interquartile range)	4 (1–8)		
Presence of aura, n (%)	0 (0)		
Headache intensity, visual analog scale (interquartile range)	7 (6–8)		
	Migraine patients with aura (N = 26)	Migraine patients without aura (N = 56)	*P* value
Age, years (±standard deviation)	29.3 (±11.7)	31.3 (±9.5)	.415
Male, n (%)	8 (30.7)	7 (12.5)	.047
Age of headache onset, years (interquartile range)	14.0 (12.0–25.7)	21.5 (15.0–33.5)	.177
Disease duration, months (interquartile range)	60 (12–180)	60 (27–120)	.825
Attack frequency per month, n (interquartile range)	2 (1–4)	4 (2–10)	.007
Headache intensity, visual analog scale (interquartile range)	6 (5–8)	7 (6–8)	.051

### 3.2. Difference in TMT between patients with EM and healthy controls

**Figure 2.**
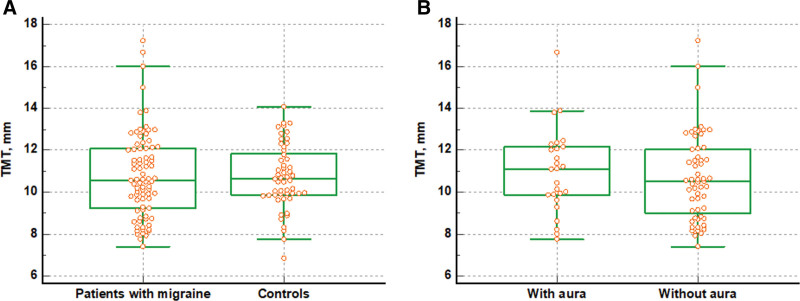
Difference in temporal muscle thickness between patients with episodic migraine and healthy controls. The figures show that the TMT is not different between patients with episodic migraine and healthy controls (10.804 vs 10.721 mm, *P* = .801) (A), nor between migraine patients with and without aura (10.994 vs 10.716 mm, *P* = .569) (B). TMT = temporal muscle thickness.

The TMT was not different between patients with EM and healthy controls (10.804 ± 2.045 mm in patients with EM vs 10.721 ± 1.547 mm in healthy controls, *P* = .801) (Fig. 2A). Furthermore, the TMT was not different according to presence of migraine aura in patients with EM (10.994 ± 2.016 mm in patients with migraine aura vs 10.716 ± 2.071 mm in those without migraine aura, *P* = .569) (Fig. 2B).

### 3.3. Correlation analysis between TMT and clinical characteristics

We could not find any significant correlations between TMT and clinical characteristics, including age (*r* = 0.042, *P* = .708), age of onset (*r* = 0.110, *P* = .359), duration of migraine (*r* = −0.081, *P* = .509), headache intensity (*r* = 0.004, *P* = .976), or headache frequency (*r* = −0.084, *P* = .501) in patients with EM.

## 4. Discussion

This study was the first to investigate sarcopenia in patients with migraine. However, we did not confirm a relationship between patients with migraine and sarcopenia using TMT. Furthermore, no correlations were identified between TMT and various clinical characteristics in patients with migraine.

Brain MRI provides the opportunity to explore and identify neuroinflammation in vivo allowing for the characterization of specific inflammatory elements, including vascular permeability and macrophage or microglia activity.^[9]^ Experimental models have demonstrated the confirmation of macrophage-mediated inflammation through the application of ultra-small superparamagnetic iron oxide particle-enhanced MRI.^[25]^ Dynamic contrast-enhanced MRI has been developed and validated, unveiling novel prospects to investigate blood–brain barrier permeability in migraine.^[25–27]^ Prior MRI studies have yielded inconsistent results depending on study design, the presence of aura, and the elapsed time since the onset of headache attacks. In cohorts where MRI was conducted during the interictal state or where participants with aura were not classified, no differences in vascular permeability parameters were observed.^[27,28]^ Efforts to establish a correlation between changes observed in cell microscopy directly associated with systemic inflammation and MRI studies continue. Studies have consistently linked systemic inflammation to decreased cerebral gray matter, cortical, and hippocampal volumes. Furthermore, altered MR properties imply microstructural variations in cellular and molecular composition.^[29]^ Recent research has accumulated substantial evidence regarding the association between perivascular spaces on MRI and neuroinflammation.^[30]^

In this study, we observed the absence of TMT differences between the patient group and healthy controls, which may be attributed to the exclusive inclusion of patients with EM. Chronic migraine (CM) is a disabling disorder, and according to a study, the annual transformation from EM to CM is reportedly approximately 2.5%.^[31]^ Several risk factors for CM have been recognized.^[31,32]^ These factors include an increased migraine attack frequency, excessive use of acute migraine medications, depression, and lifestyle elements such as stress, high caffeine intake, and obesity.^[32–34]^ The pathophysiology and the mechanisms that result in transformation are not fully understood. There is substantial evidence proposing inflammation and central sensitization as pivotal mechanisms in the evolutionary process of CM.^[32]^ Prolonged release of CGRP can induce peripheral sensitization, likely attributed to the release of inflammatory mediators from nerve endings and immune system cells.^[32,35]^ Key cytokines such as TNF-α, IL-1β, and IL-6 have been implicated in the process leading to neurogenic inflammation.^[36]^ Elevated levels of CGRP result in continuous activation of C-fibers (which store CGRP) and Aδ-fibers (containing CGRP receptors), potentially leading to the production and release of inflammatory cytokines not only in the dura but also in neuronal cell bodies localized in the trigeminal ganglion.^[37]^ Nevertheless, it is worth noting that this study focused solely on the analysis of newly diagnosed EM, which may have reduced the influence of neuroinflammation. To prove our hypothesis, future sarcopenia studies in patients with EM are needed.

Cortical spreading depression (CSD) is widely recognized as the underlying mechanism of migraine aura.^[38]^ In animal models, it has been observed that macrophages may release cytokines such as IL-1β and TNF-α, which are reported to be elevated after CSD.^[39]^ This phenomenon has also been confirmed in patients with migraine with aura.^[40]^ A study utilizing functional imaging demonstrated that migraine with aura is associated with neuroimmune activation and neuroinflammation, supporting a potential link between CSD and glial activation.^[41]^ The uptake levels of the ligand for the 18-kDa translocator protein, a marker of glial activation, were measured. The uptake signal increased in nociceptive processing areas, such as the thalamus, primary/secondary somatosensory cortices, and insular cortices, as well as in regions associated with the occurrence of CSD.^[41]^ In another study targeting individuals with migraine with aura, a persistent extra-axial inflammatory signal was identified in the meninges and calvarial bone overlaying the occipital lobe in those experiencing visual aura.^[42]^ This finding indicates the presence of extensive neuroinflammation in both the cortex and parameningeal tissues of migraine patients with aura.^[42]^ Hence, we aimed to investigate potential differences in neuroinflammation based on the presence of aura, employing novel neuroimaging techniques. However, in this study, we did not observe differences in TMT, a marker of sarcopenia, based on the presence or absence of aura.

Previously reported research has indicated that, in addition to abdominal skeletal muscles, craniofacial skeletal muscles, such as the cross-sectional area of the skeletal muscle mass at the cervical level, are valuable in assessing the extent of muscle mass loss.^[43]^ In a study conducted in patients with brain metastasis, a strong correlation between TMT and lumbar skeletal muscles was revealed.^[20]^ Additionally, in a study involving patients with head trauma, the total psoas muscle area was directly correlated with TMT.^[18]^ We selected the temporal muscle for estimating skeletal muscle mass on routine brain MRI because it is one of the few muscles that is depicted in its entirety on these scans.^[44]^ The measurement of TMT is advantageous compared to measuring other skeletal muscle parameters as it is conducted more easily and quickly. The strengths of this method are further underscored by a previous study demonstrating a high level of agreement between inter- and intra-rater reliability.^[20]^ Recently, the TMT has been shown to exhibit a negative correlation with age and the lowest oxygen saturation in patients with obstructive sleep apnea. This finding supports the feasibility of utilizing conventional brain MRI to assess sarcopenia in patients with various neurological disorders.

This study was the first to investigate sarcopenia in patients with migraine. Additionally, sarcopenia was analyzed depending on the presence or absence of migraine aura. Moreover, we enrolled a relatively large number of patients with migraine. However, this study had some limitations. First, this study was conducted at a single center with a retrospective design. Secondly, we used TMT measurement to assess sarcopenia, which has the advantage of being able to utilize routine brain MRI. However, there could be a bias because manual segmentation was used to assess TMT. The usefulness of TMT has already been proven in many previous studies, and one experienced neuroradiologist performed all measurements to decrease bias. Lastly, to prove our hypothesis, studies using several inflammatory markers collected at the time of brain MRI are needed.

There was no difference in TMT between patients with EM and healthy controls. Furthermore, no correlations were observed between TMT and clinical characteristics. These findings suggest a lack of evidence for sarcopenia in patients with EM. However, this study was the first to investigate sarcopenia in patients with migraine.

## Author contributions

**Conceptualization:** Kang Min Park.

**Data curation:** Dong Ah Lee, Ho-Joon Lee, Kang Min Park.

**Formal analysis:** Kang Min Park.

**Methodology:** Ho-Joon Lee, Jinseung Kim, Kang Min Park.

**Project administration:** Dong Ah Lee, Ho-Joon Lee, Kang Min Park.

**Software:** Ho-Joon Lee, Jinseung Kim, Kang Min Park.

**Supervision:** Kang Min Park.

**Validation:** Dong Ah Lee, Kang Min Park.

**Visualization:** Dong Ah Lee, Kang Min Park.

**Writing – original draft:** Dong Ah Lee, Ho-Joon Lee, Jinseung Kim, Kang Min Park.

**Writing – review & editing:** Dong Ah Lee, Kang Min Park.
